# Paediatric cerebral palsy in South Africa: Prevention and care gaps at hospital level

**DOI:** 10.4102/ajod.v13i0.1449

**Published:** 2024-10-30

**Authors:** Thembi J. Katangwe, Mariana Kruger, Ronald van Toorn, Jeanetta van Zyl, Sandile Ndlovu, Regan Solomons, Kirsten A. Donald

**Affiliations:** 1 Department of Pediatrics and Child Health, Faculty of Medicine and Health Sciences, Stellenbosch University, Stellenbosch, South Africa; 2 School of Psychology, University of KwaZulu-Natal, Pietermaritzburg, South Africa; 3 Department of Pediatrics and Child Health, Faculty of Health Sciences, University of Cape Town, Cape Town, South Africa; 4 Department of Neuroscience Institute, University of Cape Town, Cape Town, South Africa

**Keywords:** cerebral palsy, perinatal asphyxia, prematurity, care gaps, risk factors

## Abstract

**Background:**

Population-based data show high proportions of severe cases of cerebral palsy (CP) in resource-poor regions such as sub-Saharan Africa, where most children have potentially preventable risk factors (factors that may increase the likelihood of CP occurrence but can be mitigated through medical interventions).

**Objectives:**

This study aimed to describe the demographic and clinical profile of children living with CP accessing services at Tygerberg Hospital over a period of 10 years (2010–2020), identify the potential gaps in care (proportion of individuals in a country requiring but not receiving suboptimal or inadequate care), and comparison with a similar study at the same centre two decades ago.

**Method:**

This 10-year retrospective study investigated causes and morbidities in children with CP, attending a central hospital in the Western Cape, South Africa.

**Results:**

A total of 613 children with CP were identified. Perinatal causes were predominant, especially in 57.7% (*n* = 354) of the cohort: perinatal asphyxia (41.1%) and preterm birth (16.6%). Postnatal causes constituted 15.2% (*n* = 93), which included tuberculous meningitis (3.6%) and bacterial meningitis (3.6%). The most common complications were intellectual impairment (61.8%; *n* = 379); epilepsy (30.8%; *n* = 189) and visual impairment (54.7%; *n* = 234). A third of the cohort had severe CP, classified as Gross Motor Function Classification System IV and V (38%).

**Conclusion:**

Most of the previously documented main drivers of CP are still present and the implementation of healthcare prevention strategies remains inadequate.

**Contribution:**

This study provides longitudinal evidence to confirm that CP in a South African setting is associated with a high burden of potentially preventable causes.

## Introduction

Globally, cerebral palsy (CP) remains an important cause of motor disability. It has a reported prevalence of more than 4 per 1000 live births in low- and middle-income countries (LMICs) (Murugasen et al. 2024), compared to 1.6 per 1000 live births in high-income countries (HICs) (Katangwe et al. 2024). Aetiological differences between different regions have been documented for CP. In HICs, prematurity associated with extremely low birth weight is most frequently documented, while perinatal asphyxia and kernicterus remain prevalent causes in less-resourced settings (Hollung et al. 2018; Smithers-Sheedy 2016). Cerebral palsy registers in HICs have proven to be effective in documenting aetiological and phenotypic trends for individuals living with CP as well as providing data that led to the implementation of targeted strategies to improve prenatal, perinatal and postnatal healthcare, with a subsequent decline in CP prevalence (Johnson, Blair & Stanley 2011; Morgan et al. 2016). For many HICs, besides strengthening neuroprotective strategies for infants born prematurely, the quality of life for children living with CP has also improved (Makris, Dorstyn & Crettenden 2021).

Data paucity in LMICs including South Africa, remains a major hindrance in understanding the full spectrum of aetiologies. This directly impacts opportunities for planning preventative strategies alongside motivating for the required health workers and infrastructure to support these approaches. The estimated CP prevalence for South Africa is derived from a few studies, which have reported up to 10 per 1000 live births in certain communities (Murugasen et al. 2024). In many LMICs, perinatal asphyxia, kernicterus and central nervous system (CNS) infections (Katangwe et al. 2024) remain important CP causes. Results from the Global LMIC CP register confirm differences in aetiology of CP between LMICs and HICs (McIntyre et al. 2022). While some risk factors are common across contexts, there are important risk factors that are region-specific. Examples of such factors prevalent in sub-Saharan Africa and far less common in HIC settings include postnatal infections, such as tuberculous meningitis (TBM) and cerebral malaria (Katangwe et al. 2024).

A previous study (between 2003 and 2004) of 242 children living with CP and accessing care also at this teaching hospital found a high prevalence of perinatal insults (38% of total cases; predominantly birth asphyxia and prematurity-related complications) (Van Toorn et al. 2007). Of note was also a very high percentage of children with acquired causes of CP (21%), especially secondary to TBM and kernicterus. Therefore, it is essential to investigate the CP aetiology over time to ensure the implementation of evidence-based interventions to address preventable causes, especially as there is an increase in the medico-legal litigation against healthcare professionals for CP caused by preventable conditions.

This study therefore aims to investigate whether the risk factors and aetiologies of CP have changed over the last two decades and whether the gaps in healthcare access and specific interventions remained. The study objectives were as follows: (1) to describe the socio-demographic characteristics of children living with CP in this cohort; (2) to describe the identified risk factors for CP, ascertain the timing of the brain insult and/or injury where possible and document the physical classification and the motor function by using the Gross Motor Function Classification System (GMFCS) level; (3) to describe the most common co-morbidities experienced by children living with CP and to describe the gaps in care for children with CP at this tertiary facility; (4) to compare findings to a study performed in the same setting between 2003 and 2004 (Van Toorn et al. 2007).

## Research methods and design

### Setting and study population

This was a descriptive retrospective cohort study of 613 children (1–13 years of age) living with CP, seen in tertiary-level specialised paediatric developmental and paediatric neurology clinics between January 2010 and December 2020 at Tygerberg Hospital. The hospital is a large state-funded hospital with 302 secondary and tertiary paediatric beds, serving approximately half of the 2.4 million children requiring healthcare services in the Western Cape province, South Africa (Tygerberg Hospital 2022).

Children aged between 1 and 13 years (at the time of the data collection) who accessed paediatric neurology and neurodevelopmental services at the teaching hospital with a confirmed diagnosis of CP were included. The diagnosis fulfilled the ‘Surveillance of Cerebral Palsy in Europe and the Australian Cerebral Palsy Register’ case definition that includes the following key elements: (1) an injury to the developing brain that is non-reversible and remains non-progressive; (2) it affects movement, posture, and motor function; and (3) the insult or anomaly occurs in a developing brain (Surveillance of Cerebral Palsy in Europe [SCPE] 2000).

### Data collection

Routinely collected demographic and outcome data, populated through the Enterprise Content Management System (ECM) of the teaching hospital were reviewed. The children’s families’ socioeconomic stata were deduced from the income classification used by the Western Cape Provincial Government of South Africa to determine subsidies for public healthcare usage within the province (Western Cape Government 2022). Patients are either fully subsidised if they are unemployed or pensioners and partially subsidised according to their earnings. A full breakdown of all healthcare subsidy criteria that is used in the province can be seen in the footnote of Table 1.

**TABLE 1:** Baseline characteristics of children living with cerebral palsy at Tygerberg Hospital.

Child characteristic	%	*N* = 613	Mean	s.d.	Median	Range
**Sex**						
Male	60.4	370	-	-	-	-
Female	39.6	243	-	-	-	-
**Age (years)**						
Mean age (s.d.)	-	-	8.1	2.8	-	-
Range	-	-	-	-	-	1.0–13.0
Median age	-	-	-	-	9	-
**CP type at the time of diagnosis^a^**						
Spastic	-	-	-	-	-	-
Unilateral	16.5	101	-	-	-	-
Bilateral	-	-	-	-	-	-
Lower limbs more spastic	12.2	75	-	-	-	-
All four limbs equally spastic	31.6	194	-	-	-	-
Mixed	10.8	66	-	-	-	-
Dystonic	4.6	28	-	-	-	-
Dyskinetic	2.4	15	-	-	-	-
Athetoid	1.6	10	-	-	-	-
Triplegia	1.3	8	-	-	-	-
Ataxia	0.5	3	-	-	-	-
Evolving CP	7.0	43	-	-	-	-
CP type not stated	11.4	70	-	-	-	-
**GMFCS classification^b^**						
GMFCS I	17.0	104	-	-	-	-
GMFCS II	12.1	74	-	-	-	-
GMFCS III	5.9	36	-	-	-	-
GMFCS IV	6.0	37	-	-	-	-
GMFCS V	32.0	196	-	-	-	-
CP Not classified^c^	27.0	166	-	-	-	-
**Age at identifying children at a high risk of developing CP (weeks)**						
Mean age (s.d.)	-	-	16.4	20.9	-	-
Range	-	-	-	-	-	3–19
Median	-	-	-	-	9	-
**Socio-economic status**						
H0^d^	5.9	36	-	-	-	-
H1^e^	86.1	528	-	-	-	-
H2^f^	5.5	34	-	-	-	-
H3^g^	2.4	15	-	-	-	-

CP, cerebral palsy; GMFCS, Gross Motor Function Classification System; s.d., standard deviation.

a Choreoathetoid, dystonic, athetoid, ataxia;

b Gross Motor Function Classification System Classification (GMFCS): I Walks without limitations; II Walks with limitations; III Walks using a hand-held mobile device; IV Self-mobility with limitations, may use powered mobility; V Transported in a manual wheel chair;

c The reasons for non-classification were as follows: (1) children recorded as ‘evolving CP’; (2) no information regarding the GMFCS available at the time of diagnosis;

d Full State Susbidisation (H0);

e (Partial state subsidisation single income less than ZAR70 000 (USD 3736) per individual or ZAR100 000 (USD 5337) per family income per annum) H1:;

f (Partial state subsidization_income from ZAR70 000 (USD 3736) to ZAR100 000 (USD 5337) per single income or ZAR 250 000 (USD 13 345) to ZAR 350 000 (USD 18 680) per family income per annum (H2);

g Partial state subsidisation_income of more than ZAR 250 000 (USD 13 345) per single income or more than ZAR350 000 family income per annum (H3).

Data validation was carried out by listing all children attending the neurology and/or neurodevelopment clinics during the study period with their unique hospital record number. The unique hospital record number was thereafter used to access full patient medical records in the provincial patients electronic database named ECM for the Western Cape province. Where there were discrepancies, the paper-based patient records were retrieved for further data collection and validation.

Clinical documentation in case notes, laboratory and special investigation reports were used to determine CP aetiology. Perinatal asphyxia was defined using the 2019 American College of Gynaecology (ACOG) task force criteria on neonatal encephalopathy and CP (The American College of Obstetrics and Gynaecology 2017, 2019). Suggestive history, clinical examination, laboratory findings including auditory brainstem responses and magnetic resonance imaging (MRI) were used to confirm chronic bilirubin encephalopathy (kernicterus). Polymerase chain reaction (PCR) test results, viral cultures and neuroimaging findings confirmed congenital infection. The presence of at least one of the following (1) intraventricular haemorrhage (IVH) with or without ventriculomegaly on cranial ultrasound scan; (2) the presence of periventricular leukomalacia (PVL) on brain imaging confirmed white matter injury of prematurity.

White matter injury of prematurity is an umbrella term of all the pathologies that result in cerebral white matter injury of immature brains in preterm babies. These include germinal matrix haemorrhage-intraventricular haemorrhage (GMH-IVH), PVL, and diffuse white matter injury (Lee et al. 2017).

Definite bacterial meningitis was confirmed by the presence of a positive gram stain and/or positive bacterial culture on cerebrospinal fluid (CSF). Tuberculous meningitis was identified using the TBM research case definition (Marais et al. 2010) comprising clinical, laboratory and radiological components. Hypoglycaemic brain injury was confirmed by the presence of the following:

- Plasma CSF glucose <2 mmol/L
- T2-weighted MRI images showing bilateral white matter signal intensities in the occipital and parietal lobes.

Hypovolemic shock was confirmed by the presence of at least two of the following:

- Documented hypotension
- Evidence of fluid resuscitation and inotropic support
- Evidence of ischaemic brain injury on imaging.

Prenatal causes of CP were classified as non-progressive brain injuries that occurred in the developing brain in utero, anytime from the post-conception period to the period before birth (Bax et al. 2005). Perinatal causes were non-progressive brain injuries that occurred in the developing brain during birth. Postnatal causes were non-progressive brain injuries that occurred after birth (Bax et al. 2005). The SCPE classification system (SCPE 2000) was used to classify CP. The documented topographic classification was used as supporting evidence for classification (Bax et al. 2005). Motor function was captured using the GMFCS (Paulson & Vargus-Adams 2017).

Comorbidities were defined as disorders occurring alongside CP but may also occur independently without the CP (Hollung et al. 2020). The intellectual level of a child with CP in this cohort was determined by using the Molteno developmental screening assessment tool (Springer et al. 2022) and from that the Developmental Quotient (DQ) was calculated and recorded for each child in their files. Children without information on their DQ assessment were categorised in an ‘unknown’ group (Brown, Parikh & Patel 2020). Behavioural problems were identified from the narrative text of the hospital notes, bearing in mind challenges in the identification of behaviourally defined conditions in a child with significant neuromotor disability. A child with CP was deemed epileptic if they had two or more unprovoked seizures more than 24 h apart, with or without documented electroencephalogram and neuroimaging results and confirmation of chronic anti-epileptic usage.

The vision was categorised into five main groups:

- Normal vision
- Some other visual impairment (strabismus, refractive errors, gaze dysfunction, visual field defects)
- Confirmed cerebral cortical visual impairment
- Normal light perception – can fixate and follow
- Blindness.

The hearing was categorised into four main groups:

- Normal hearing
- Some hearing impairment (use of hearing aids, conductive hearing loss, and sensorineural hearing loss)
- Confirmed deafness
- No data available.

Allied health services were defined as healthcare professionals excluding medical professionals (aHPCSA 2018). Examples of healthcare services include physiotherapy, occupational therapy, speech and language therapy, audiology, nutrition and dietetics, social work and health education. A child was deemed to have accessed allied health services if their records documented attendance of at least four of any of these services regardless of the time frame.

### Statistical strategy

Descriptive statistics were used to analyse demographic data, clinical subtypes and motor function (GMFCS). Categorical variables were reported using absolute values (n) and reciprocal percentages (%), corrected to one decimal point. Medians and interquartile ranges (IQRs) were calculated for numerical values, assuming normal distribution. All data were analysed using STATA version 16.0 (StataCorp 2019). This statistical package was recommended by our statistics expert as it offers improved functions that assist with data cleaning as well as having a wide range of statistical techniques, with advanced features for managing missing data, a commonality in retrospective studies.

### Ethical considerations

Ethical approval to conduct this study was obtained from the Stellenbosch University, Health Research Ethics Committee (No. S21/10/195).

## Results

**FIGURE 1:**
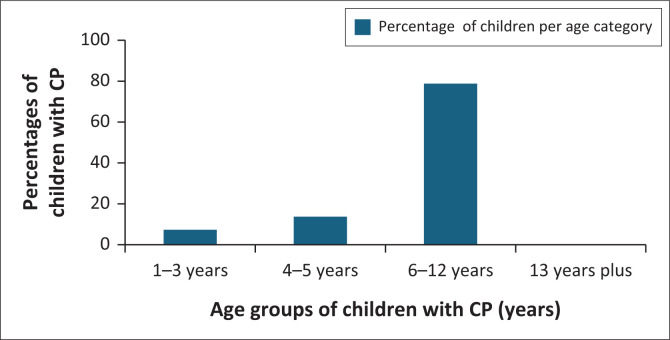
Age distribution of children living with cerebral palsy at Tygerberg Hospital.

A total of 613 children were identified with CP. The median age at study inclusion was 9 years (IQR 6–10 years). Most of these children were of school-going age (78.8%; *n* = 483) (see Figure 1). The majority were males (60.4%; *n* = 370) and the median age of identifying at-risk children for CP was 9 weeks (IQR 3–19 weeks).

### Characteristics of children living with cerebral palsy

Spastic CP was the most common type of CP in this cohort (60.4%; *n* = 370), followed by mixed CP type (10.8%; *n* = 66). Within this spastic group, bilateral spastic CP was the predominant CP type (*n* = 370; 60.4%), with a third having equal involvement of all four limbs (*n* = 194; 31.6%) and 12.3% (*n* = 75) having more lower limb involvement. Unilateral spastic CP was present in 16.5% (*n* = 101). Some of the other CP types included dystonic CP (4.6%; *n* = 28), dyskinetic CP (2.4%; *n* = 15) and ataxic CP (0.5%; *n* = 3) (Table 1).

The majority of the cohort had severe motor function, GMFCS IV and V (38.0%; *n* = 233). The annual income for most of the families in this cohort was < ZAR100 000 (<USD [United States Dollar] 5337) per annum (86.1%; *n* = 528), with a small proportion (5.9%; *n* = 36) depending solely on the pension income of grandparents (Table 1).

### Causes of cerebral palsy

The perinatal period was the most vulnerable time for CP to occur (57.7%; *n* = 354) in this cohort, with perinatal asphyxia (41.1%; *n* = 252) and white matter injury of prematurity (16.6%; *n* = 102) as the important causes.

Perinatal asphyxia occurred more in boys, with a male-to-female ratio of 1.8:1. Bacterial meningitis (3.6%; *n* = 22), TBM (3.6%; *n* = 22), postnatal ischaemic episodes (3.1%; *n* = 19) and kernicterus (2.4%; *n* = 15) were the top causes of postnatal CP (Table 2).

**TABLE 2:** Reported cerebral palsy etiological factors at Tygerberg Hospital.

Characteristic	Current study (*N* = 613)		2002–2003 study (*N* = 242)	
	%	*n*	%	*n*
**Prenatal**				
Structural brain anomalies^a^	8.2	50	15.7	38
Cryptogenic	2.1	13	2.1	13
Prenatal stroke	3.3	20	5.7	14
Congenital infections^b^	5.2	32	1.7	4
Prenatal toxin exposure^c^	0.3	2	1.7	4
Prenatal causes not documented or other prenatal causes	3.8	23	1.2	3
**Perinatal**				
Perinatal asphyxia	41.1	252	17.3	42
White matter injury of prematurity	16.6	102	17.7	43
**Postnatal**				
Bacterial meningitis	3.6	22	6.2	15
Tuberculosis meningitis	3.6	22	9.5	23
Hypoglycaemia	0.5	3	-	-
Kernicterus	2.4	15	2.1	5
Postnatal ischaemic episodes^d^	3.1	19	-	-
Non accidental injuries and/or head trauma	0.5	3	-	-
Epileptic encephalopathies	1.0	6	-	-
Others	0.5	3	-	-
Cause of CP undocumented	4.2	26	-	-

Source: Van Toorn, R., Laughton, B., Van Zyl, N., Doets, L. & Elsinger, F., 2007, ‘Aetiology of cerebral palsy in children presenting at Tygerberg Hospital’, *South African Journal of Child Health* 1(2), 74–77, viewed n.d., from http://www.sajch.org.za/index.php/SAJCH/article/view/47/8

CP, cerebral palsy.

a Adam Oliver Syndrome, posterior fossa malformations, neuro tube defects, migration disorders, septro-optic dysplasia;

b Cytomegalovirus (43.4%), Syphilis (9.4%), Human Immunodeficiency virus (30.2%), Tuberculosis (9.4), Rubella (3.8%), pneumonia (3.8%);

c Prenatal Methamphetamine and alcohol exposure;

d Dyke Davidoff Mason Syndrome, Hemiconvulsion-Hemiplegia Epilepsy Syndrome, Moya Moya Syndrome, Status epilepticus, Acute Life-Threatening Events.

### A comparison of cerebral palsy causes in this study with those from the previous study (2003–2004)

A previous CP study (2003–2004) conducted at this facility found that white matter of prematurity (17.7%) was the most reported CP risk factor followed by perinatal asphyxia (17.3%). Bacterial meningitis contributed 6.2% (15 cases out of 242), while TBM and kernicterus contributed 9.5% and 2.0%, respectively (Van Toorn et al. 2007).

### Cerebral palsy comorbidities

For intellectual impairment, 78.8% (483) of the children had a documented DQ score of which 61.8% (*n* = 379) were deemed to have intellectual impairment. More than a third, namely, 37.7% (*n* = 231) had severe intellectual impairment. Epilepsy was identified in 30.8% (*n* = 189) of the children while visual impairment was detected in 54.7% (*n* = 234) of the children. The most important causes of visual impairment in this cohort were a group of visual perception disorders, refractive errors, gaze dysfunctions and visual field defects (29.5%; *n* = 181). The second important complication was cerebral cortical visual impairment (10.7%; *n* = 66). Overall, 8.0%; *n* = 49 of the total sample was classified as blind with the following specifically documented causes: congenital cytomegalovirus infection 0.7% (*n* = 4); structural brain anomalies 2.0% (*n* = 12); bacterial meningitis 0.3% (*n* = 2) and TBM 0.1% (*n* = 1). For those with epilepsy, hypoxic-ischaemic encephalopathy (16.6%; *n* = 102) was the most documented presumed cause for their epilepsy (Table 3).

**TABLE 3:** Comorbidities and access to cerebral palsy related healthcare services.

Associated impairments	%	*n*
**Intellectual impairment (*N* = 613)**		
No impairment (DQ > 85 or so described)^a^	17.0	104
Mild- moderate (DQ 51–85 or so described)	24.1	148
Severe (DQ < 50 or so described)	37.7	231
No information available	21.2	130
**Behavioural problems documented^b^ (*N* = 613)**		
Aggression, self-injury, anxiety, impulsivity	7.0	43
No behavioural problems noted (Attempt made by the clinician to document information on child’s behaviour)	39.0	239
ASD^c^	1.0	6
ADHD^d^	0.7	4
No behavioural information documented	52.3	321
**Epilepsy (*N* = 613)**		
Present	30.8	189
Absent	56.3	345
No information given	12.8	79
**Visual impairment (*N* = 613)**		
None	11.4	70
Some other visual impairment (strabismus, refractive errors, gaze dysfunction, visual field defects)	29.5	181
Confirmed cerebral cortical visual impairment	10.8	66
Normal light perception- can fixate and follow	6.2	38
Blind^e^	8.0	49
Visual status not commented on	34.1	209
**Hearing impairment (*N* = 613)**		
No impairment	32.6	200
Some hearing loss (*Use of hearing aids, conductive hearing loss, sensorineural hearing loss, unilateral hearing loss)*	21.4	131
Bilateral deafness	3.8	23
No data available	42.3	259
**Frequently prescribed allied health services^f^ (*N* = 613)**		
Occupational therapy	68.8	422
Physiotherapy	27.4	168
Speech therapy	3.8	23
**Schooling (*N* = 613)**		
Special schooling	10.4	63
Mainstream schooling	5.8	36
Special care center	5.8	36
Waiting list for special schooling	5.0	30
Not in school^g^	33.2	204
No schooling data documented^h^	39.8	244
**Mobility assistive devices for GMFCS IV-V % (*N* = 233)**		
Wheelchair with full postural support (Madiba to go buggy)	52.4	122
Wheelchair with flexible postural support (sam active)	4.7	11
Wheelchair that allows independent mobility (sully active)	22.7	53
Other devices	18.4	43
No mobility assistive device^i^	1.7	4

a Developmental Quotient (DQ);

b McDermott, S., Coker, A.L., Mai, S., Krishna swami, S., Nagle, R.J., Barnett-Queen, L.L. et al., 1996, ‘A population-based analysis of behavior problems in children with cerebral palsy’, *Journal of Pediatric Psychology* 21(3), 447–463. Available from: https://pubmed.ncbi.nlm.nih.gov/8935244/;

c Autistic Spectrum Disorders (ASD);

d Attention Deficit Hyperactivity Disorder (ADHD);

e The causes of the blindness included: Congenital Cytomegalovirus Infection 0.7% (*n* = 4); Structural brain anomalies 2.0% (*n* = 12); Bacterial Meningitis 0.3 % (*n* = 2); Tuberculosis Meningitis 0.1% (*n* = 1); cause of blindness undocumented 4.9 % (*n* = 30);

f Each child accessed more than one allied health service during the 10 years under study;

g The two frequently recorded reasons for children not being enrolled in any schooling institution were that these children were classified as Gross Motor Function Classification System (GMFCS) V with multiple co-morbidities including visual impairment and children with CP that were less than 5 years of age;

h Reasons for unknown schooling status: no information available, lost to follow up, transferred out before schooling age, family relocated;

i Children were either on the waiting list to receive assistive devices or the devices had not been requested.

### Access to services

Occupational therapy (68.8%; *n* = 422), physiotherapy (27.4%; *n* = 168) and speech therapy (17.9%; *n* = 110) were the frequently accessed allied health services. Of the 196 children with GMFCS V, 58.7% (*n* = 115) had mobility assistive devices. Wheelchairs with full postural support were the most prescribed assistive devices in this group (13.4%; *n* = 82). Other prescribed assistive devices included wheelchairs that allow independent mobility (1.3%; *n* = 8) and wheelchairs with flexible postural support (1.1%; *n* = 7).

School-aged children comprised 86.0% (*n* = 527) of this cohort; however, only 24.9% (*n* = 131) had schooling information documented in their case notes. The recorded schooling information indicated that 49.6% (*n* = 65) were not in school, 11.5% (*n* = 15) were enrolled in mainstream education, while 19.4% (*n* = 26) were enrolled in schools for learners with special educational needs. Of this cohort, 46.2% (*n* = 30) were on the waiting list for schools for learners with special educational needs, while mere 9.9% (*n* = 13) were in Special Care Centres (non-profit organisations that care for children with severe disabilities in South Africa).

## Discussion

To the best of our knowledge, this is the first study in South Africa that re-investigated the causes and morbidities of children living with CP after more than a decade. Our findings show that the proportion of severe cases of CP remains very high and that the majority of children have potentially preventable risk factors such as perinatal asphyxia, white matter injury of prematurity, kernicterus and bacterial and/or tuberculosis (TB) meningitis. The fact that the main drivers of CP have remained unchanged suggests that healthcare prevention strategies remain inadequate.

The estimated prevalence of perinatal asphyxia in South Africa ranges from 2.3 to 26.5 per 1000 live births (Stofberg et al. 2020). The wide range in CP prevalence can be explained by the complexity of this condition and the numerous diagnostic criteria being utilised. At this institution, the in-born rate for severe neonatal encephalopathy was 5.5/1000 in singleton live-born babies (9/1000 rate for live-born deliveries ≥ 36 weeks) (Adams, Mason & Gebhardt 2022). The ‘Saving Mothers and Babies Report on Perinatal Care’, 2012–2013 & 2017–2019 (National Perinatal Morbidity and Mortality Committee 2013, 2019), found that nearly 50% of modifiable factors for perinatal asphyxia are associated with healthcare provider behaviour and that more than 50% of perinatal asphyxia cases occur at the district level (National Perinatal Morbidity and Mortality Committee 2013, 2019). Potentially modifiable factors include failure to identify foetal distress (with and without foetal monitoring), delay in perinatal referral to a secondary or tertiary hospital, and inappropriate management of prolonged second stage of labour. The World Health Organization (WHO) guidelines have recommended 30–75 min as a safe decision to delivery time interval (Apako et al. 2023). Unfortunately, few studies report the decision to delivery intervals in resource-poor countries and this WHO-recommended interval is often not achievable. At Tygerberg Hospital, the average decision to incision was 1 h 40 min. This finding coupled with an average bed occupancy of 102% in the emergency maternity centre (Adams et al. 2022) highlights the challenges faced in poorly resourced settings.

The Hypoxic Ischaemic Encephalopathy (HIE) incidence at this facility is high (Adams et al. 2022) when compared to incidences in HICs. A Denmark neonatal unit reported an incidence of 1.64/1000 live births (Garne et al. 2018). However, other LMIC neonatal units, Nigeria for example, recorded even higher neonatal incidences of HIE, 28/1000 live births (Ugwu, Abedi & Ugwu 2012). Our study’s findings, therefore, affirm the need to scrutinise the drivers of perinatal asphyxia within Tygerberg, especially its referring hospitals and earmark these as possible areas for implementing targeted preventative strategies.

In randomised clinical trials, therapeutic hypothermia (TH) has been shown to reduce disability in neonates with moderate-to-severe HIE in HICs (Chawla 2024). Therapeutic hypothermia has since 2008 become an established mode of therapy at this facility and is currently being offered at most tertiary hospitals in South Africa. However, most of the perinatal asphyxia cases are reported at the district level, and cooling is not offered to all asphyxiated neonates because of delayed presentation, equipment unavailability and severe clinical illness. This hinders objective assessments of the intervention’s overall impact. In addition, there is insufficient evidence to recommend TH at the district level (i.e., very few studies from resource-poor settings) and currently, its use is restricted to tertiary centres.

The institution has seen a 50% improvement in the survival of peri-viable neonates (gestational age 20–26 weeks) (Dormohamed & Van Wyk 2022). The use of non-invasive neonatal ventilation and surfactant administration have immensely contributed to this improved survival rate. Therefore, this tertiary hospital is seeing the dual streams of improving the survival of preterm infants, similar to the trends described in HICs, while still struggling to prevent perinatal asphyxia, just like many other LMICs (Ramaswamy et al. 2021). For example, the transport system for getting women with foetal distress into the hospital where they can get appropriate care is poor (Gebhardt 2016). This means that although the facilities can provide good care if access to the right kind of care is not timeous (i.e. basic transport infrastructure and early recognition of foetal distress), irreversible brain injury remains a high risk. As such, a relatively low proportion of neonates with perinatal asphyxia may be accessing TH.

As with the previous study, the overrepresentation (15%) of acquired post-neonatal CP cases is concerning. Despite the proven therapeutic benefits of phototherapy for preventing kernicterus, LMIC continues to report high rates of avoidable exchange transfusions, as well as bilirubin-induced mortality and neurodevelopmental disorders. Interventions implemented at Tygerberg Hospital include phototherapy and exchange transfusion protocols, training of personnel and innovative technology such as non-invasive transcutaneous bilirubin monitors. The latter became available at this facility and peripheral clinics in 2013 and has alleviated the sampling stress endured by newborns, mitigated iatrogenic anaemia and expedited the turnaround time for obtaining results and providing treatment.

Tuberculosis is a major health problem in the Western Cape of South Africa, which explain the high contribution of TB meningitis-related CP cases in both studies. Unfortunately, the prevalence of childhood TBM at this tertiary facility has remained largely unchanged over the last decade because of social factors such as poverty and overcrowding as well as the negative impact brought about by a global Bacillus Calmette-Guerin (BCG) vaccine shortage and the coronavirus disease-19 (COVID-19) pandemic.

The median age of flagging babies at high risk of developing CP was 9 weeks. This early identification of at-risk neonates is a result of having obstetric and neonatal units at the same facility that allows for a structured follow-up service (King et al. 2022). The lower proportion of severe cases of CP (GMFCS IV and V; 38.0%) at this facility as compared to other LMICs where the proportion of children with GMFCS IV and V motor disability is more commonly over 50% (Jahan et al. 2021) may be because of the integrated neonatal, high-risk, and neurology services that allow for early CP diagnosis and follow-up all within the same facility. This is a unique finding for Tygerberg Hospital as most tertiary centres in LMICs have disaggregated neonatal and chronic paediatric follow up services where CP is most likely to be identified.

Even though Tygerberg Hospital has had successes in improving neonatal care over the years, other major gaps in healthcare services still exist. Firstly, the lack of progress in perinatal care for labouring mothers, evidenced by the persistence of perinatal asphyxia as a cause of CP in this facility for over a decade. Secondly, this facility does not offer adequate allied health services for children with CP. Our findings show that only 27.4% of these children access physiotherapy at this tertiary facility. The reason behind this low access is likely to be multifactorial, including factors such as the financial burden of transporting non-ambulatory children to the hospital by families, inadequate staffing, high turnover at the facility resulting in sparse appointments and resulting perceived limited effectiveness (Narain & Mathye 2023). Although older children placed in special schools within the province may access in-house therapies at the schools and therefore the overall access to therapies may be slightly higher than we report here, only 10.4% of the children in this cohort were placed in these schools suggesting this as a relatively small potential limitation. Thirdly, overall, only 27.0% of children in the total cohort were placed in some form of schooling facility within the province. This study highlights the gap that exists in the provision of education services for children with CP in South Africa. Even though access to special schooling in South Africa is better than in many resource-poor countries (Jahan et al. 2021), placing children with disabilities in schools remains a significant challenge because of lengthy waiting lists and limited places in schools in the region. Lastly, another gap is the poor access to appropriate mobility assistive devices for children with CP. This study highlighted that even though, almost all the non-ambulatory children (GMFCS IV–V), had a prescribed mobility assistive device, only 52.1% of these children had their seating appropriately addressed with postural wheelchairs. The reasons for this gap include limited availability and accessibility of devices, high costs (e.g., electrical wheelchairs) inadequate assessment and referral procedures as well as limited access to allied specialists services. The rest of the children in this severe category were prescribed other forms of mobility assistive devices. Local studies in South Africa have identified gaps in the implementation of policies related to these devices, including delays in assessment, referrals, device prescription as well as maintenance (Visagie et al. 2022).

The findings from this study indicate that the country is facing insufficient healthcare prevention strategies. Therefore, to mitigate some of these challenges, there is a need to focus on further improvements in obstetric and neonatal services. To objectively determine and address the main challenges within these crucial service domains, there is a need for the implementation or strengthening of the following specific healthcare prevention strategies: firstly, programmes to prevent preterm labour and provision of adequate care for premature babies; secondly, improved maternal health by ensuring access to prenatal care, nutrition and health education for pregnant woman; thirdly, infectious disease prevention (TB and HIV [Human Immunodeficiency Virus] screening); fourthly, skilled healthcare birth attendance to reduce the risk of birth asphyxia; fifthly, improved postnatal care (TH, screening for jaundice, support for new mother); sixthly, healthcare worker training; and lastly, data collection, and research to allow the establishment of a CP registry.

This study’s results indicate the Western Cape province in South Africa may still have insufficient healthcare prevention strategies. To mitigate the causes of acquired CP, both obstetric and neonatal services should improve. The Western Cape Department of Health should assess the quality of obstetric and neonatal care, and implement strategies to address the identified gaps in healthcare provision. Case-control studies within the obstetric and neonatal units should be conducted to identify specific risk factors and patient outcomes to assist in recommendations of targeted CP preventative strategies. Health Economics studies that assess the effectiveness of the current healthcare system and its long-term cost implications can also better motivate improvements in the current state of the health system so that quality healthcare is offered across the board.

This study was biased in many ways by being a retrospective study. Firstly, the study was biased towards children with severe CP as the study was conducted at a tertiary facility. As a result, some children with the milder severity CP may have been missed. Secondly, only recorded information in the children’s hospital records was considered, as such some information was missing or incomplete. Thirdly, there may have been CP classification misclassification because many of the documented clinical classification metrics were reported in the older Topographic CP classification system, according to the standard of care at the time. Therefore, to mitigate some of these biases, we ensured matching patient identification data from the clinical records in the neurology and neurodevelopmental clinics, physical patient files in store at the clinic as well as the electronic records on ECM. As such, we upheld the currently endorsed SCPE CP definition guidelines to re-assign the appropriate physical classification for all the children.

We therefore recommend that future studies are prospective and population-based to ensure accurate CP calculation as well as result generalisation. Despite these limitations, this study is one of the few reports that has attempted to highlight the gaps in the care offered to children with CP in LMICs. In addition, the study has also compared findings to those of a similar study that was done at the institution almost two decades ago, allowing comment on this population over the span of 20 years.

## Conclusion

Major findings revealed a high proportion of severe CP cases, with 38% classified as GMFCS IV and V. Perinatal causes, primarily perinatal asphyxia (41.1%) and preterm birth (16.6%) were predominant, while postnatal causes included TBM and bacterial meningitis. The study concluded that preventable risk factors for CP remain prevalent, indicating insufficient healthcare prevention strategies. It is clear that most of the previously documented main drivers of CP are still present and that implementation of healthcare prevention strategies remains inadequate. Therefore, future studies need to be case-control studies that will provide an in-depth understanding of the specific gaps within obstetric and neonatal care to better inform policy and the implementation of targeted CP preventative strategies.
